# Salinity gradients alter root-zone soil microbiome structure and nitrogen-related functional potential in alfalfa (*Medicago sativa* L.): a pot experiment

**DOI:** 10.3389/fpls.2026.1753229

**Published:** 2026-02-13

**Authors:** Mingzi Lv, Delong Tian, Guoshuai Wang, Chenli Hou, Ting Fan, Weiping Li

**Affiliations:** 1 School of Energy and Environment, Inner Mongolia University of Science and Technology, Baotou, China; 2 Collaborative Innovation Center of Autonomous Region for Ecological Protection and Comprehensive Utilization in the Inner Mongolia Section of the Yellow River Basin, Baotou, China; 3 Yinshanbeilu Grassland Eco-hydrology National Observation and Research Station, China Institute of Water Resources and Hydropower Research, Beijing, China; 4 Institute of Water Resources for Pastoral Area, Ministry of Water Resources, Hohhot, Inner Mongolia, China

**Keywords:** alfalfa, metagenomics, nitrogen cycling, root-zone soil microbiome, salinity, soil salinization

## Abstract

**Introduction:**

Soil salinization constrains agricultural sustainability in arid and semi-arid regions. This study examined integrated soil–plant–microbe responses of alfalfa (*Medicago sativa* L.) to a salinity gradient.

**Methods:**

A pot experiment was conducted with control, low-, and moderate-salinity treatments. Root-zone soil and plants were sampled to measure soil EC, pH, and inorganic nitrogen forms, and to assess plant growth traits. Shotgun metagenomics was used to characterize microbial community composition and metagenome-inferred functional potential.

**Results:**

Salinity increased soil EC and pH and altered inorganic nitrogen forms, with higher NO_3_^-^-N under moderate salinity and lower NH_4_^+^-N under salinity compared with the control. Plant height peaked under low salinity, whereas fresh and dry biomass decreased under both salinity treatments. Microbial β-diversity differed among treatments, while α-diversity showed limited responses. Functional annotations indicated treatment-associated trends in nitrogen- and stress-related categories and KEGG level 3 pathways; however, most differences were not significant after FDR correction.

**Discussion:**

This integrative root-zone assessment links salinity-driven soil chemistry changes with alfalfa performance and suggests coordinated shifts in soil chemistry, microbiome structure, and plant growth under salinity stress.

## 1 Introduction

Soil salinization is an escalating environmental challenge that constrains agricultural productivity and sustainability, particularly in arid and semi-arid regions (Litalien and Zeebe, 2020). Excess soluble salts impose osmotic stress and ion imbalance on plants and can reduce yield and degrade soil quality (Xiao and Zhou, 2023). Saline–alkaline soils occupy large areas globally, and salinity represents a major constraint in northern China (including Inner Mongolia) (China Agricultural Network, 2024), where forage production and ecological restoration are strategic priorities (Zhang et al., 2023; Zhu et al., 2022; Yang et al., 2022).

Salt-tolerant forage species and phytoremediation provide sustainable approaches for rehabilitating salt-affected soils while maintaining productivity (Fan et al., 2017; Lin et al., 2019). *Medicago sativa* L. (alfalfa) is a perennial legume with moderate salt tolerance and dual value for forage production and soil improvement (Liu et al., 2019; Zhao et al., 2020; Wang et al., 2021; Li et al., 2024). Its deep rooting system can improve soil structure and infiltration, while symbiotic biological nitrogen fixation increases soil N inputs and supports soil fertility (Zhang et al., 2022, Zhang et al., 2023; Liu et al., 2023). Root exudates may further modify the chemical microenvironment near roots, potentially influencing nutrient availability and ion mobility (Vives-Peris et al., 2019).

Soil microorganisms are key components of the root-associated microecosystem and influence nutrient cycling and stress-related processes that can affect plant performance under salinity (Fan et al., 2011; Oulen, 2022). Salinity may reshape root-zone community assembly through altered ion composition, nutrient status, and root-derived substrates (Yue et al., 2020; Yang et al., 2020), and salt-tolerant taxa have been proposed to support plant tolerance via osmoprotection and antioxidant-related functions (Liang et al., 2019). However, in forage-legume systems, it remains unclear how salinity-driven changes in root-zone soil chemistry co-occur with shifts in microbial community structure and metagenome-inferred functional potential, and how these patterns relate to plant growth and nitrogen status. Recent reviews and meta-analyses synthesize broadly consistent salinity-driven shifts in root-associated microbiome assembly and potential functional responses across diverse plants and ecosystems. These syntheses highlight the need for integrative designs that link soil chemistry with plant and microbiome traits (Zhang et al., 2024).

Most prior salinity–microbiome studies in crops have relied on 16S rRNA amplicon sequencing, which describes community composition but provides limited resolution for functional genes and pathways. In contrast, shotgun metagenomics improves taxonomic resolution and enables inference of functional potential, offering a framework to evaluate nitrogen-related and stress-response functions that may vary along salinity gradients. This integrative perspective is particularly relevant for saline agroecosystems in northern China, where improving forage productivity and soil rehabilitation is a practical priority.

In this pot experiment, we integrated soil physicochemical analyses, plant performance measurements, and shotgun metagenomic sequencing to examine soil–plant–microbe responses to a salinity gradient in the root-zone. We tested the following hypotheses: (1) increasing salinity significantly shifts microbial community composition (β-diversity) more strongly than overall α-diversity; (2) salinity is associated with changes in metagenome-inferred nitrogen-related and stress-response functional potential; and (3) salinity-driven variation in soil properties (e.g., EC, pH, inorganic N forms) covaries with microbiome structure/functional potential and alfalfa growth traits. Specifically, we aimed to: (1) quantify treatment effects of salinity on soil properties and alfalfa growth; (2) characterize treatment-associated patterns in microbial diversity, community composition, and functional profiles; and (3) evaluate relationships among soil chemistry, microbiome features, and plant traits using correlation-based analyses. Importantly, the novelty of this work lies in the integrated assessment framework that links salinity-driven root-zone soil chemistry changes with alfalfa performance and shotgun metagenome-inferred microbiome structure and functional potential in a controlled pot system, rather than in proposing new nitrogen-cycling mechanisms.

## 2 Materials and methods

### 2.1 Study area description

The experiment was conducted in 2024 in Saihan District, Hohhot, Inner Mongolia Autonomous Region, China (40°48′26″ N, 111°44′48″ E). The region has a temperate continental monsoon climate, with an average annual temperature ranging from 6.3 °C to 7.7 °C and mean annual precipitation of 138.2 mm. Annual evaporation varies from 2,030 mm to 2,700 mm, and the frost-free period lasts approximately 133–144 days. The soil type was classified as chestnut soil. To document the pre-treatment (baseline) conditions shared by all pots, soil properties were measured prior to salinity application: electrical conductivity (EC) 0.27 mS cm^-^¹, pH 7.89, nitrate nitrogen (NO_3_^-^–N) 1.02 mg kg^-^¹, total nitrogen (TN) 980.48 mg kg^-^¹, and total phosphorus (TP) 550.09 mg kg^-^¹.

### 2.2 Experimental design and sampling methods

A pot experiment was performed using alfalfa (*Medicago sativa* L.) cultivar ‘Algonquin’. Field-collected soil was passed through a 4-mm sieve, homogenized, and air-dried under shade prior to use. Alfalfa seeds were germinated in plug trays, and seedlings were transplanted into pots four weeks after emergence. Each plastic pot (26.6 cm diameter, 24.5 cm height) was filled with 7.5 kg of sieved soil. After thorough irrigation, 20 seedlings were transplanted into each pot.

**Figure 1:**
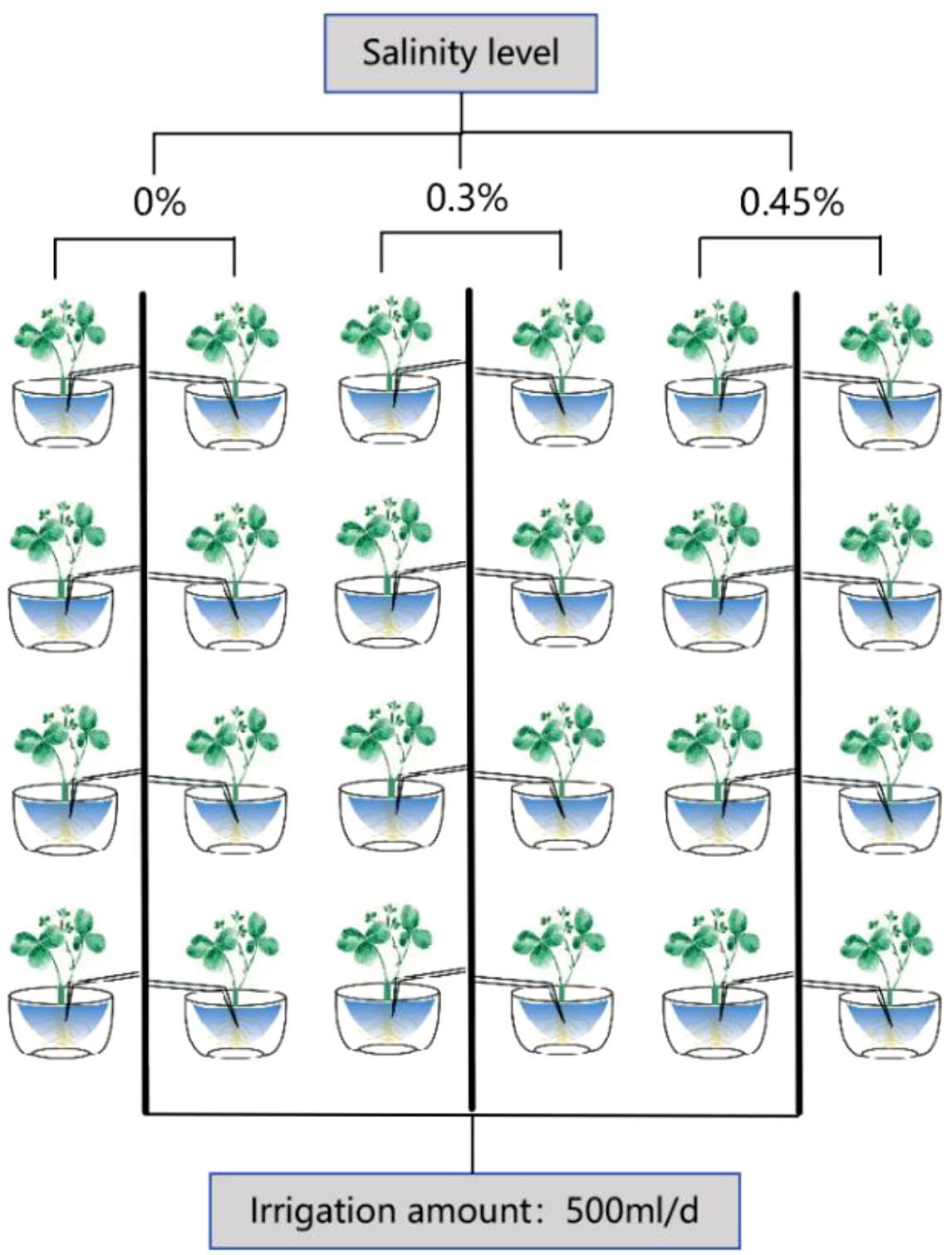
Schematic diagram of the pot experiment setup and salinity treatments applied in this study. Plants were subjected to three salinity levels (0%, 0.3%, and 0.45%) with a constant irrigation amount of 500 mL per day. Treatment codes were defined as follows: CK, 0% salinity (control); LS, 0.3% salinity (low salinity); MS, 0.45% salinity (moderate salinity).

Salinity was imposed by mixing a neutral salt blend of NaCl and Na_2_SO_4_ (1:1, w/w) into the soil to achieve two target levels: low salinity (LS, 3.0 g kg^-^¹ dry soil) and moderate salinity (MS, 4.5 g kg^-^¹ dry soil), with a non-saline control (CK). The NaCl + Na_2_SO_4_ mixture was used because chloride and sulfate salts commonly co-occur in saline soils, making mixed neutral salts more representative than NaCl alone (Reginato et al., 2021). The two application rates (3.0 and 4.5 g kg^-^¹) were selected to impose mild and moderate salinity stress while avoiding excessively severe conditions frequently used in pot studies (Hou et al., 2022; Irakoze et al., 2021). Salts were thoroughly mixed into the soil once at the start of the treatment, after which all pots received the same irrigation regime. All pots were irrigated with 500 mL of deionized water per day; the control (CK) received the same water volume as the salinity treatments. Salinity treatments started on 17 June 2024 (at transplanting stage) and were maintained until harvest on 30 September 2024 (105 days; **Figure 1**).

At harvest, both plant and soil samples were collected. Surface debris, litter, and gravel were removed prior to sampling. In this study, “root-zone soil” refers to soil collected adjacent to the visible root system within each pot. Root-zone soil was obtained by coring soil within approximately 3 cm of the roots. We did not physically separate a strictly defined rhizosphere fraction (i.e., soil tightly adhering to the root surface recovered by washing); therefore, the microbial profiles represent communities from the operationally defined root-zone soil compartment rather than strictly defined rhizosphere soil. Soil from each pot was divided into two subsamples: one was placed into 25-mL centrifuge tubes, preserved on dry ice, and transported for metagenomic sequencing; the other was sealed in plastic bags for measurements of soil pH, electrical conductivity (EC), NO_3_^-^-N, NH_4_^+^-N, and total nitrogen (TN).

### 2.3 Plant sample and soil physicochemical property measurements

#### 2.3.1 Plant samples

At the end of the alfalfa growing season (September 30, 2024), plant samples were harvested from each pot. Plant height was measured using a ruler from the soil surface to the tip of the tallest shoot. Plants were then separated into shoots and roots, gently rinsed with deionized water to remove adhering soil particles, and blotted dry with paper towels. Fresh biomass was recorded immediately after sampling. Subsequently, plant samples were oven-dried at 65 °C for 48 h until constant weight was achieved. Dry biomass was determined using an analytical balance (Model JY502, Shanghai Puchun Measure Instrument Co., Ltd., China).

#### 2.3.2 Soil physicochemical properties

Soil samples were taken destructively at the end of the alfalfa growing season to measure soil pH, EC, nitrate nitrogen, ammonium nitrogen and total nitrogen. Soil physicochemical properties were analyzed by a certified third-party laboratory through the Science Compass analytical platform (China), following national or industry standard methods. Soil nitrate nitrogen (NO_3_^-^-N) and ammonium nitrogen (NH_4_^+^-N) were determined using a continuous flow analyzer in accordance with the forestry industry standard LY/T 1228-2015 (Determination of nitrogen in forest soils). Soil electrical conductivity (EC) was measured using a conductivity meter following the national environmental standard HJ 802-2016.Soil pH was measured using a pH meter (ST300, OHAUS, USA) in a soil-to-water suspension at a ratio of 1:2.5 (w/v). For total nitrogen (TN) determination, soil samples were ground to pass through a 100-mesh sieve, and TN content was measured using an elemental analyzer following standard procedures.

### 2.4 Soil DNA extraction and metagenomic sequencing

#### 2.4.1 DNA extraction, library preparation, and sequencing

Microbial DNA was extracted from soil samples using the E.Z.N.A.^®^ Stool DNA Kit (Omega Bio-Tek, USA) following the manufacturer’s protocol. Shotgun metagenomic libraries were constructed and sequenced at Shanghai LingEn Biotechnology Co., Ltd. (Shanghai, China). For each sample, approximately 1 μg of genomic DNA was fragmented using a Covaris S220 Focused Ultrasonicator (Woburn, MA, USA) to obtain ~ 450 bp fragments. Sequencing was performed on the Illumina NovaSeq 6000 platform (paired-end 150 bp mode).

Raw reads were quality filtered using Trimmomatic (Bolger et al., 2014) to remove adapters and low-quality sequences. High-quality reads were mapped to the host genome using BWA-MEM (v0.7.17) with parameters –M –k 32 –t 16. Reads aligned to the host genome or identified as low-quality were removed, and the remaining clean reads were used for downstream analyses.

Taxonomic classification was performed using Kraken2 (Wood and Salzberg, 2014) against the NCBI RefSeq database (release 90), which includes bacterial, archaeal, fungal, viral, protist, and algal genomes. Relative abundances were refined using Bracken (Lu et al., 2017) to estimate taxonomic composition at domain-to-species levels.

#### 2.4.2 Metagenomic *de novo* assembly, gene prediction, and annotation

The data from each sample, after quality control, were assembled into contigs using the MegaHit software (Li et al., 2015) (parameters: –min-contig-len 500). The assembled sequences were then subjected to open reading frame (ORF) prediction using CD-HIT (Fu et al., 2012) (parameters: -n 9 -c 0.95 -G 0 -M 0 -d 0 -aS 0.9 -r 1), resulting in a unique gene set. The longest sequence in each cluster was considered as the representative sequence of each gene in the unique gene set. To calculate the gene abundance across all samples, the Salmon software (Patro et al., 2017) was used to obtain the number of reads for each gene. To quantify metagenome-inferred gene abundance, quality-controlled reads were quantified against the non-redundant gene catalog using Salmon (Patro et al., 2017). Gene abundance (Ab(S)) was estimated as a length-normalized measure of read support for each gene across samples. Briefly, reads uniquely mapped to a gene contributed to Ab(U), whereas multi-mapped reads were apportioned among candidate genes using a coefficient (Co) proportional to their unique-mapping support, following Li et al. (2014). Thus, Ab(S) = Ab(U) + Ab(M), where Ab(M) represents the contribution from multi-mapped reads after proportional allocation. Full equations and complete variable definitions are provided in Supplementary Methods to improve readability.

To annotate the protein sequences, BLASTX was used to search the unique gene set against the KEGG database. Based on the soil sample KO results, annotated genes were used to map pathways in the KEGG database, generating pathway maps for each sample that indicated specific functions and pathways. Raw data were deposited into the NCBI Sequence Read Archive (SRA) database (Accession Numbers: PRJNA1399466).

### 2.5 Statistical analysis

**Figure 2:**
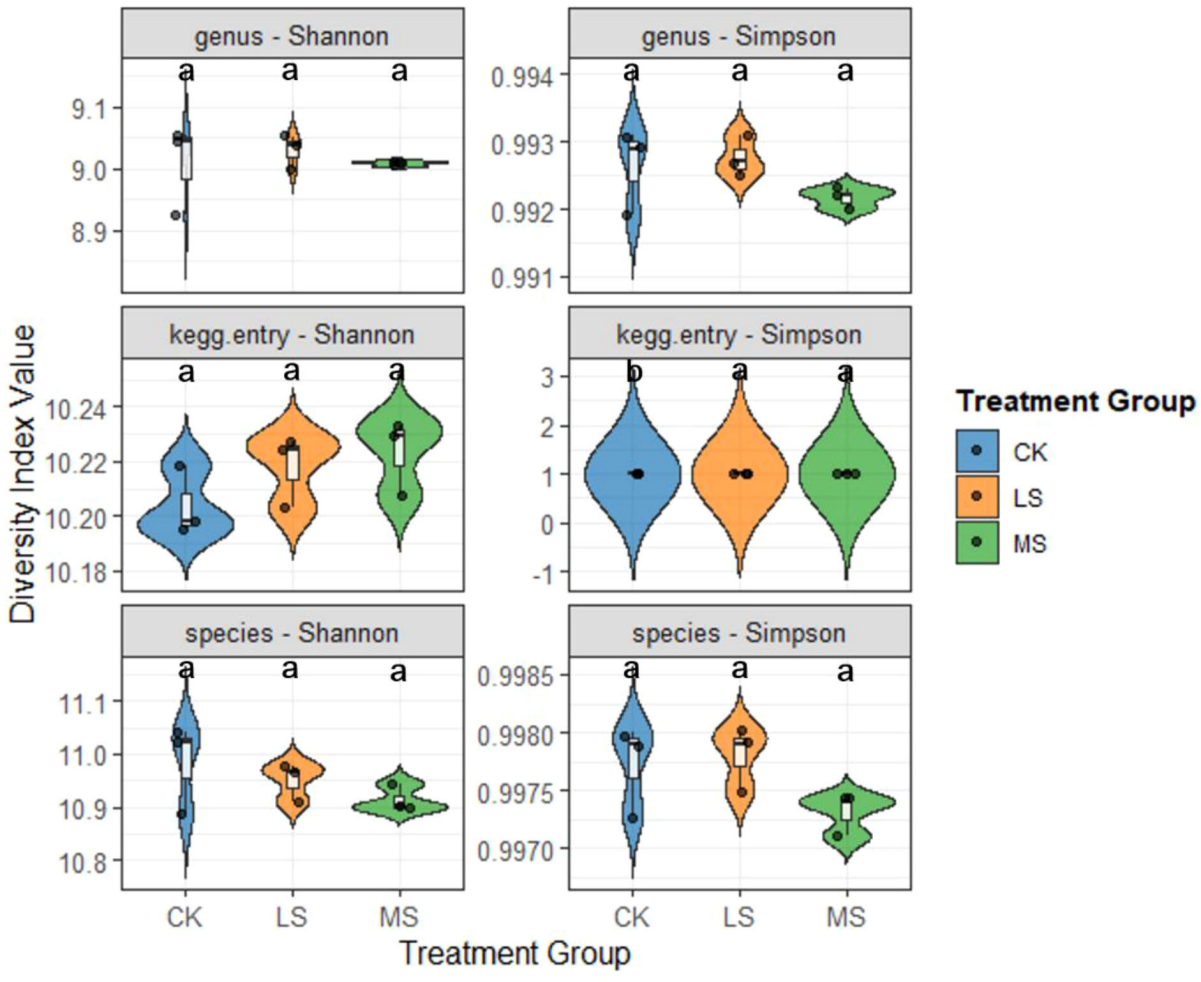
Alpha diversity of root-zone soil microbial profiles across salinity treatments. Shannon and Simpson indices were calculated at the genus, species, and KEGG-entry levels in CK, LS, and MS. Violin plots show distributions across replicates (n = 3 per treatment) with embedded boxplots (median and interquartile range). Different letters denote significant differences among treatments (one-way ANOVA with Tukey’s HSD, *p* < 0.05).

All statistical analyses were performed in R (v4.4.3). Data were assessed for normality using the Shapiro–Wilk test. For soil physicochemical properties and plant growth traits, differences among treatments (CK, LS, and MS) were evaluated using one-way ANOVA followed by Tukey**’**s HSD for ***post hoc*** comparisons. For metagenome-derived relative abundance data (e.g., dominant genera and functional categories/pathways), Kruskal–Wallis tests were applied, followed by Dunn**’**s ***post hoc*** tests for pairwise comparisons. P values from multiple comparisons were adjusted using the Benjamini–Hochberg false discovery rate (FDR) procedure. Unless stated otherwise, statistical significance was accepted at FDR-adjusted *p* < 0.05 after FDR correction, or at *p* < 0.05 for single tests. Microbial α-diversity indices (Shannon and Simpson) were compared among treatments using one-way ANOVA followed by Tukey**’**s HSD (as shown in **Figure 2**). Microbial β-diversity was assessed using principal coordinates analysis (PCoA) based on Bray–Curtis dissimilarities, and treatment effects on community composition were tested using PERMANOVA (e.g., adonis2). PERMANOVA results are reported with effect sizes (R²) and permutation-based P values. Given the low biological replication (n = 3 per treatment), statistical power—particularly for multivariate and metagenome-inferred functional comparisons—is limited; therefore, ordination patterns and functional results are interpreted cautiously, and trend-level patterns are explicitly identified where appropriate. Plots were generated in R using ggplot2 and related packages. Heatmaps and additional visualizations were rendered using the Lingbo MicroClass visualization platform.

## 3 Results

### 3.1 Effects of salinity on soil physicochemical properties

**Figure 3:**
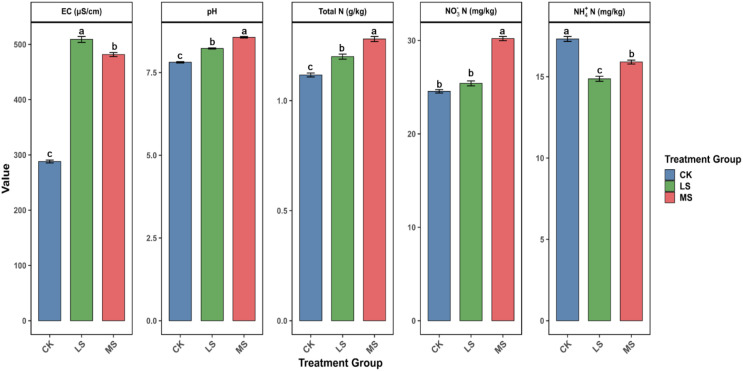
Soil physicochemical properties and inorganic nitrogen forms in root-zone soil under different salinity treatments. Electrical conductivity (EC), pH, total nitrogen (TN), nitrate (NO_3_^-^-N), and ammonium (NH_4_^+^-N) were measured at harvest in CK (control), LS (low salinity), and MS (moderate salinity) treatments. Bars indicate mean ± SE (n = 3). Different letters denote significant differences among treatments (one-way ANOVA with Tukey’s HSD, *p* < 0.05).

As shown in **Figure 3**, soil physicochemical properties at harvest differed among treatments. Soil EC increased under salinity relative to the control (approximately 290 μS cm^-^¹ in CK vs. 480–510 μS cm^-^¹ under salinity), with LS showing higher EC than MS (*p* < 0.05). Soil pH increased along the salinity gradient (CK< LS < MS), and all pairwise differences were significant (*p* < 0.05).

Salinity also altered soil nitrogen status. TN was higher under salinity than in the control (*p* < 0.05). NO_3_^-^-N increased under moderate salinity compared with CK and LS (*p* < 0.05), whereas CK and LS did not differ. In contrast, NH_4_^+^-N decreased under both salinity treatments relative to CK (*p* < 0.05), with comparable levels in LS and MS.

### 3.2 Effects of salinity on alfalfa growth

**Figure 4:**
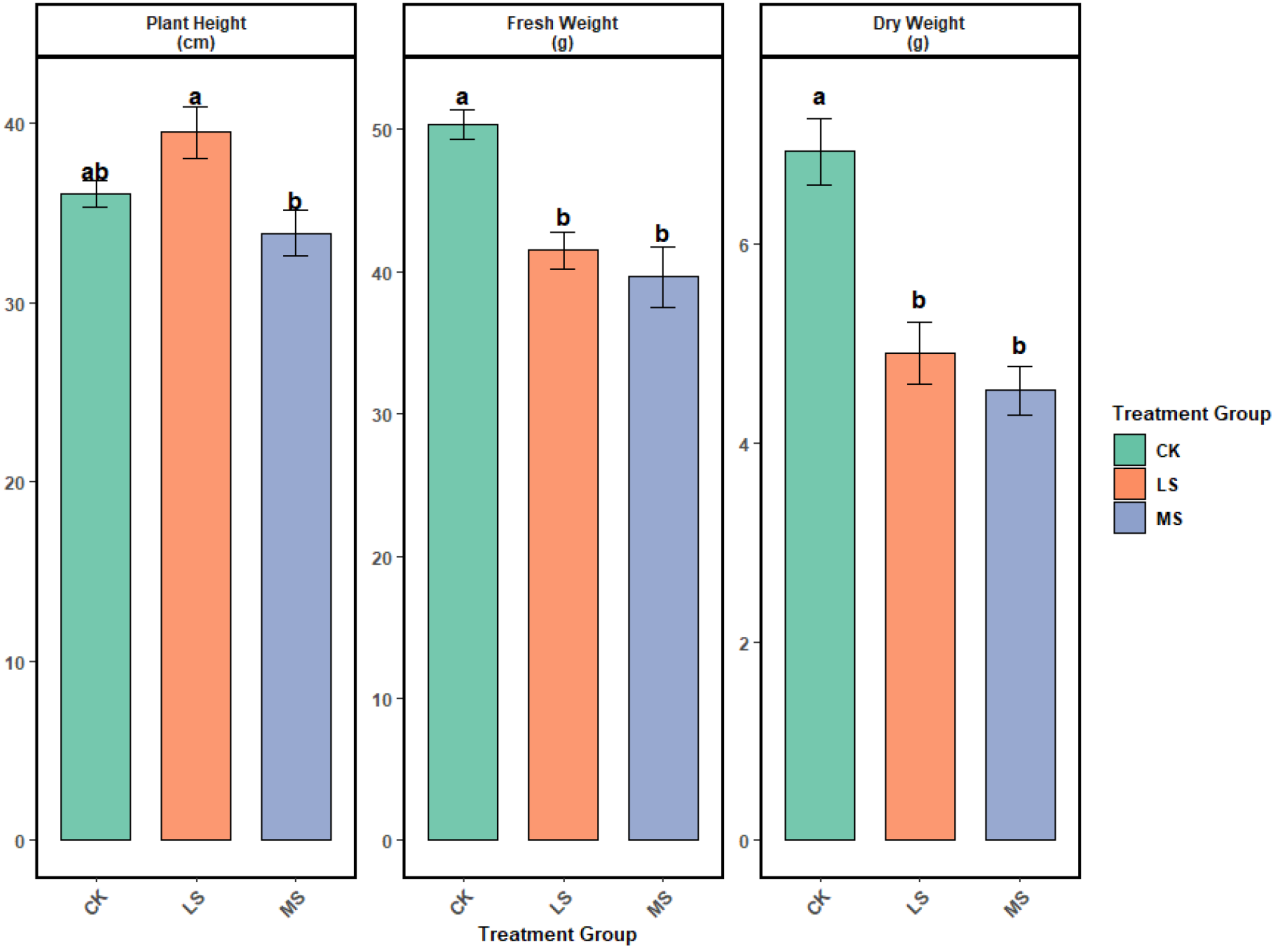
P Alfalfa growth traits under different salinity treatments at harvest. Plant height, fresh weight, and dry weight were measured in CK (control), LS (low salinity), and MS (moderate salinity). Bars indicate mean ± SE (n = 3). Different letters denote significant differences among treatments (one-way ANOVA with Tukey’s HSD, *p* < 0.05).

As shown in **Figure 4**, alfalfa growth responses differed among treatments at harvest. Plant height peaked under low salinity and was significantly higher than under moderate salinity (*p* < 0.05), whereas differences between the control and the two salinity treatments were not significant. In contrast, both fresh biomass and dry matter accumulation were significantly reduced under low and moderate salinity compared with the control (*p* < 0.05), with no significant difference between the two salinity levels. Together, these results indicate that mild salinity was associated with a height increase without a corresponding increase in biomass, while overall biomass production was sensitive to salinity stress.

### 3.3 Effects of salinity on the alfalfa soil microbial community

#### 3.3.1 α-diversity analysis

Alpha diversity was assessed using Shannon and Simpson indices at taxonomic (genus and species) and functional (KEGG entry) levels (**Figure 2**). Overall, α-diversity showed limited responses to salinity. Shannon diversity did not differ among treatments across genus, species, or KEGG-entry profiles (*p* > 0.05), and Simpson indices at the genus and species levels also showed no significant treatment effects (*p* > 0.05). In contrast, the KEGG-entry Simpson index differed between the control and the salinity treatments (*p* < 0.05), suggesting a salinity-associated shift in functional dominance patterns. Given the low replication (n = 3 per treatment), these α-diversity results are interpreted cautiously.

#### 3.3.2 Soil community composition

**Figure 5:**
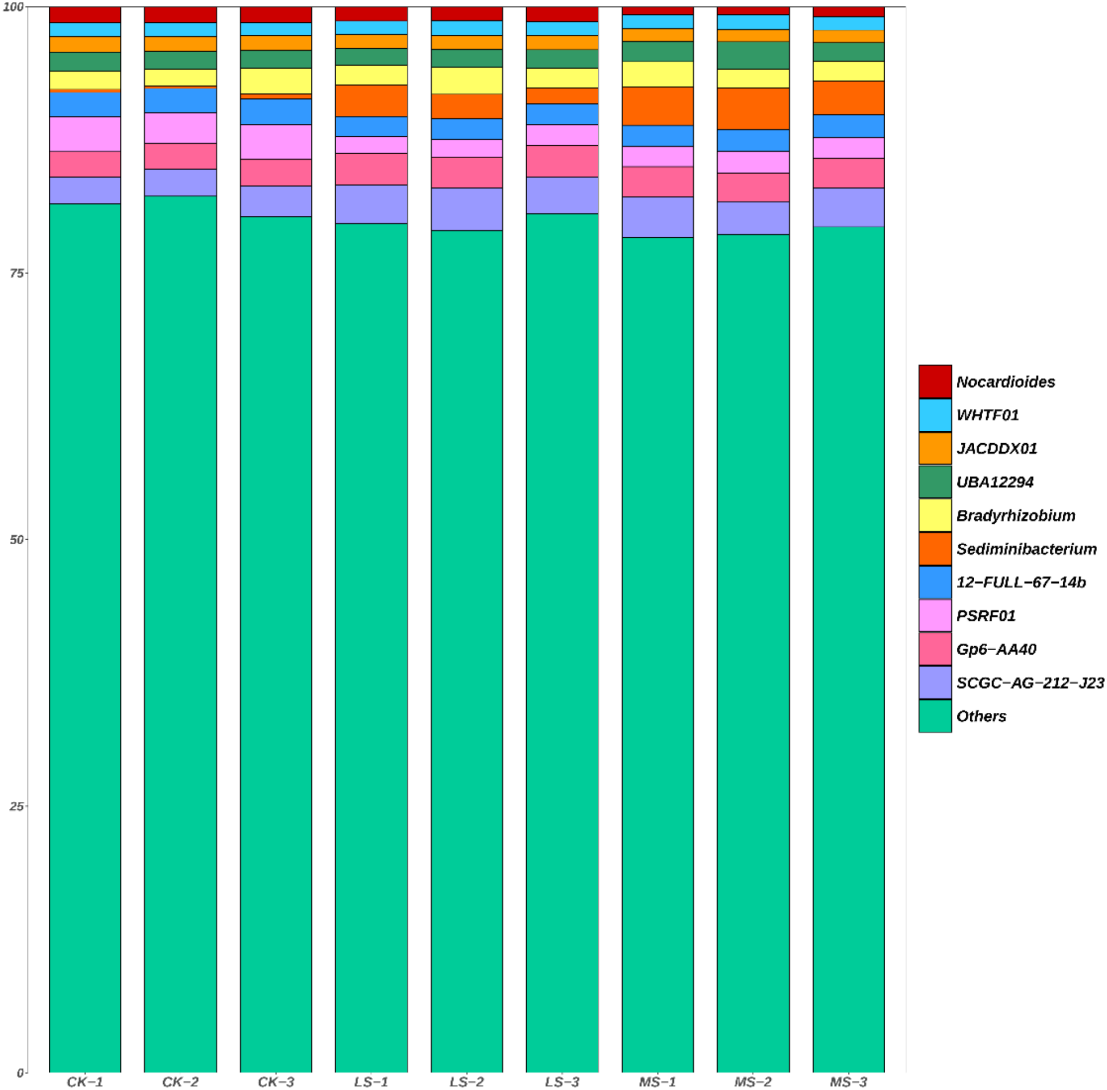
Genus-level taxonomic composition of the root-zone soil microbiome across salinity treatments. Stacked bar plots show the relative abundances of the 10 most abundant genera in each sample from CK (control), LS (low salinity), and MS (moderate salinity). “Others” represents genera outside the top 10. Each bar represents one biological replicate (n = 3 per treatment).

As shown in **Figure 5**, genus-level community composition differed among treatments. Across all samples, “Others” (genera outside the top 10) accounted for most of the relative abundance, indicating that the community was composed of many lower-abundance genera rather than being dominated by only a few taxa. Among the dominant genera, *Nocardioides* ranged from 1.47–1.56% in CK and from 0.76–0.97% in MS, whereas *Bradyrhizobium* ranged from 1.58–1.67% in CK and increased to 2.38–2.51% under salinity treatments. *Sediminibacterium* ranged from 3.41% in CK to 3.87% in MS. To formally evaluate treatment effects on dominant genera, Kruskal–Wallis tests followed by Dunn’s *post hoc* tests with FDR correction were performed. After FDR correction, none of the top 10 genera differed significantly among treatments (*p* > 0.05), although some pairwise comparisons showed nominal differences prior to correction.

#### 3.3.3 β-diversity analysis

**Figure 6:**
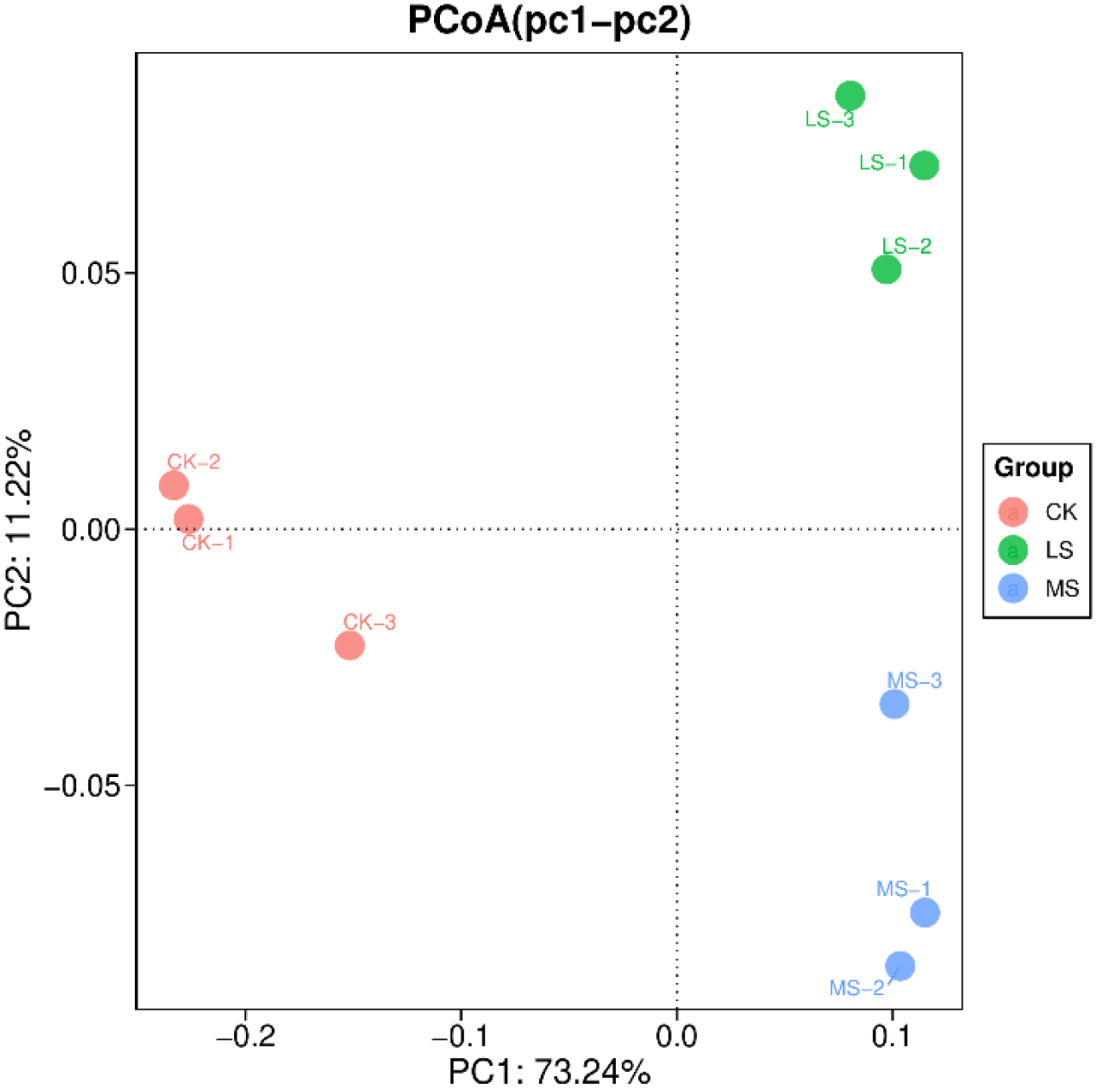
Principal coordinates analysis (PCoA) of microbial community composition based on Bray–Curtis dissimilarities. Points represent CK (control), LS (low salinity), and MS (moderate salinity) samples. The first two axes (PC1 and PC2) explain 73.24% and 11.22% of the total variation, respectively.

As shown in **Figure 6**, principal coordinates analysis (PCoA) based on Bray–Curtis dissimilarities suggested treatment-associated shifts in microbial community composition. The first two axes (PC1 and PC2) explained 73.24% and 11.22% of the total variation, respectively. CK samples tended to cluster toward negative values on PC1, whereas LS and MS samples were positioned toward positive values, consistent with compositional differences along the salinity gradient. CK replicates showed relatively tight clustering, while LS and MS replicates were more dispersed, suggesting increased compositional variability under salinity. Treatment effects on community composition were evaluated using PERMANOVA (Bray–Curtis), and effect sizes (R²). Given the low replication (n = 3 per treatment), these ordination results are interpreted cautiously as evidence of treatment-associated compositional shifts rather than definitive separation.

### 3.4 Effects of salinity on the nitrogen cycling species composition of alfalfa soil microbes

**Figure 7:**
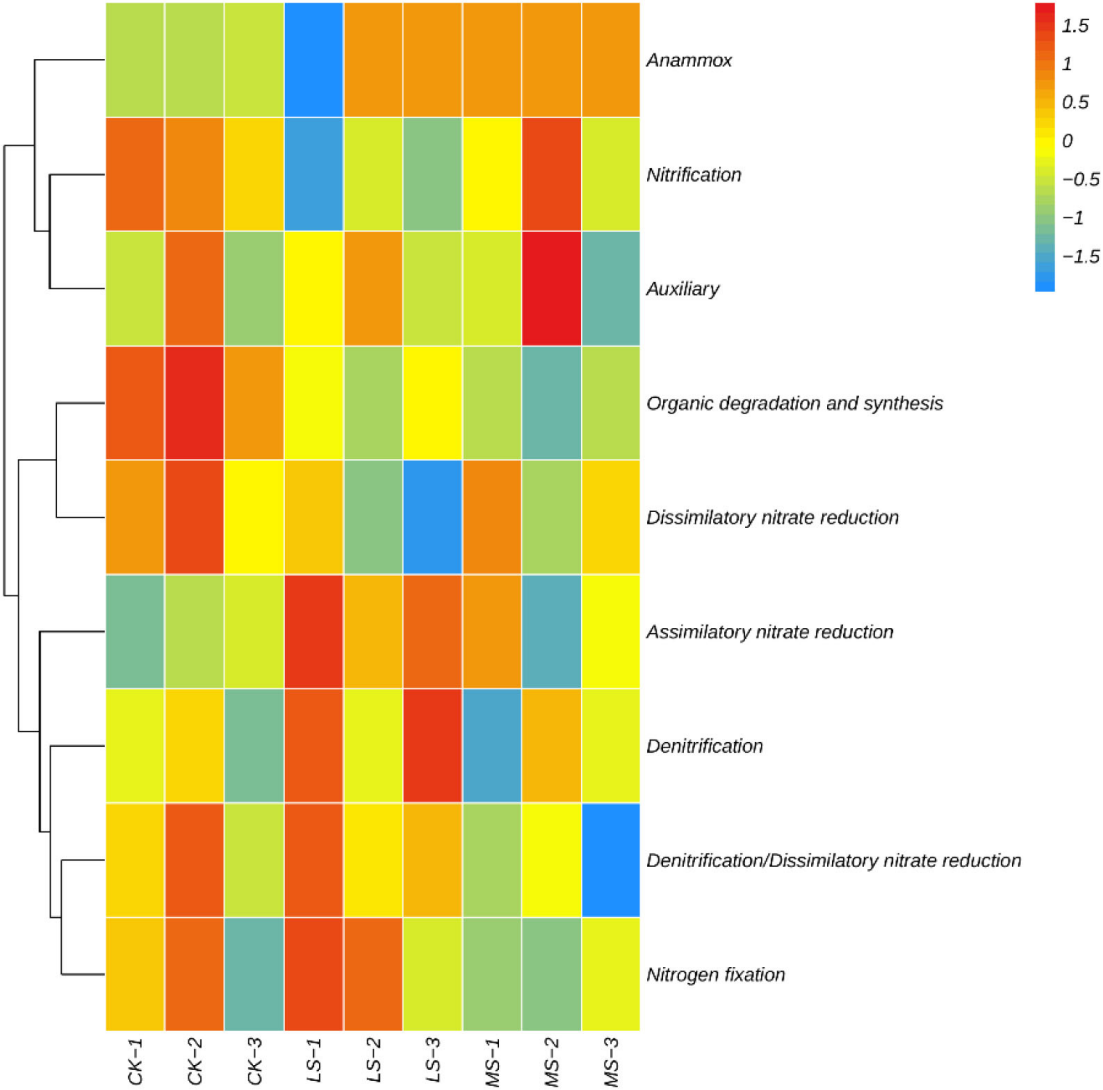
Heatmap of nitrogen cycling–related functional categories across salinity treatments. Values are row-wise z-score–normalized relative abundances (each category scaled to its across-sample mean) to visualize exploratory patterns among samples (CK, LS, MS; n = 3 per treatment). Note: Statistical tests on non-normalized values (Kruskal–Wallis with Dunn’s test and FDR correction) did not detect significant between-treatment differences (FDR-adjusted *p* > 0.05).

As shown in **Figure 7**, nitrogen cycling–related functional profiles exhibited treatment-associated trend-level variation based on row-wise z-score–normalized relative abundances. Overall, several functional groups displayed heterogeneous enrichment/depletion patterns across replicates, with the most apparent variability observed in nitrate reduction–related functions and auxiliary nitrogen metabolism, whereas some categories (e.g., organic degradation/synthesis and anammox) showed comparatively stable or low signals across treatments. To formally evaluate treatment effects, Kruskal–Wallis tests followed by Dunn’s *post hoc* tests with FDR correction were performed on the underlying (non–z-score) relative abundance values. After FDR correction, no nitrogen cycling functional category differed significantly among treatments (FDR-adjusted *p* > 0.05). Therefore, **Figure 7** is presented to visualize exploratory, relative patterning among samples rather than confirmatory, statistically supported directional changes.

### 3.5 Effects of salinity on changes in KEGG metabolic pathways in alfalfa soil microbes

**Figure 8:**
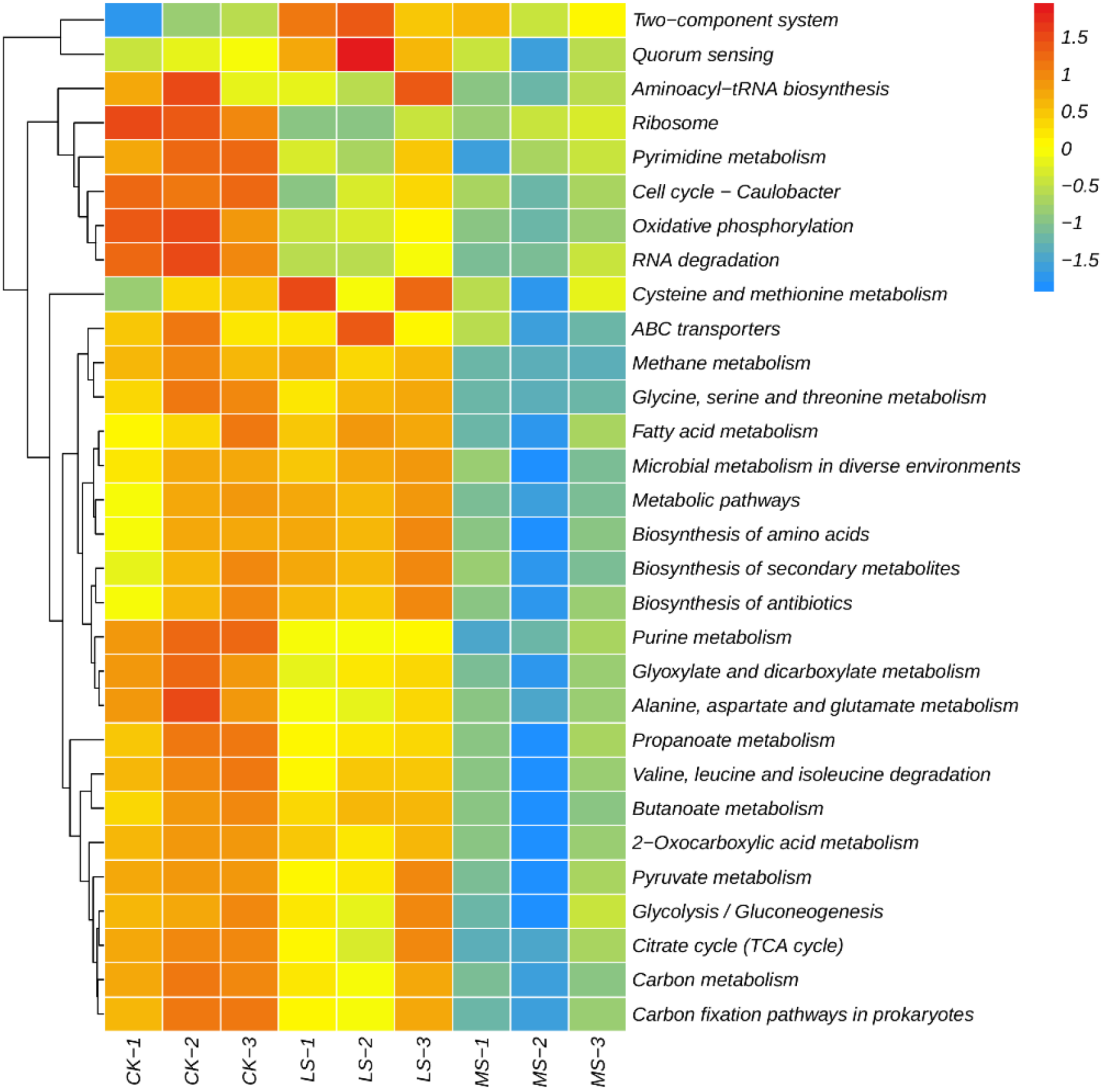
Heatmap of KEGG level 3 pathways across salinity treatments. Values are row-wise z-score–normalized relative abundances (each pathway scaled to its across-sample mean) to visualize exploratory patterns among samples (CK, LS, MS; n = 3 per treatment). Note: Statistical tests on non-normalized values with FDR correction indicated that most pathways were not significant (FDR-adjusted *p* > 0.05).

Based on the KEGG level 3 pathway heatmap (**Figure 8**) generated from row-wise z-score–normalized relative abundances, microbial functional profiles showed treatment-associated trend-level patterning along the salinity gradient. Overall, pathways related to core carbon/energy metabolism tended to show relatively higher z-scores in CK and lower z-scores under salinity, particularly in MS, whereas LS often displayed intermediate profiles. In contrast, pathways associated with environmental sensing/regulation and transport functions showed heterogeneous responses across replicates, with some categories exhibiting relatively higher z-scores in LS than in CK and MS. To evaluate treatment effects statistically, pathway-level differences were tested using Kruskal–Wallis tests followed by Dunn’s *post hoc* tests with Benjamini–Hochberg FDR correction on the underlying (non–z-score) relative abundance values. After FDR correction, most pathways were not significant (FDR-adjusted *p* > 0.05), indicating that **Figure 8** is presented primarily to visualize coordinated, exploratory trends in functional potential rather than confirmatory between-treatment differences.

### 3.6 Mantel test for correlation between microbial function and alfalfa physiological response

**Figure 9:**
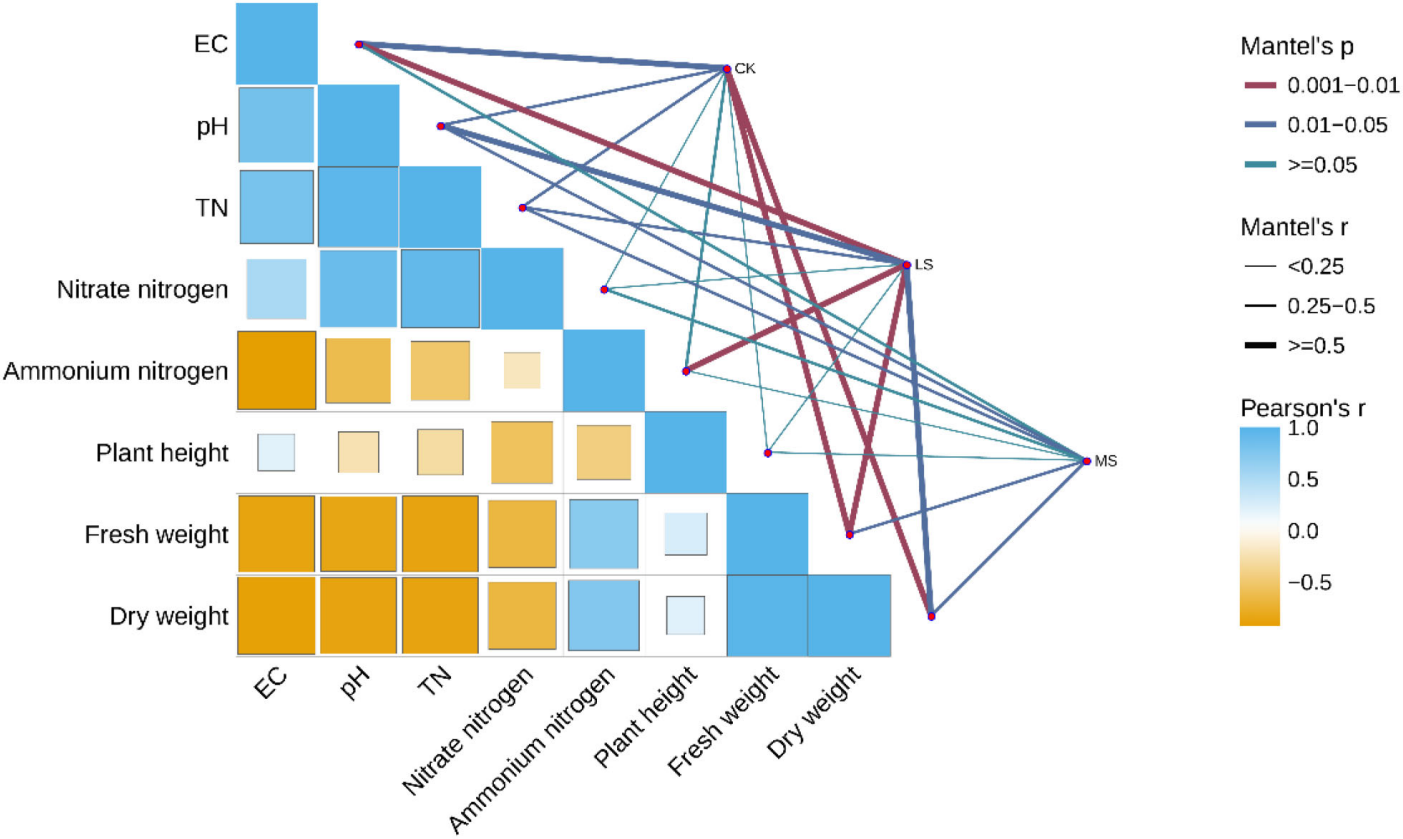
Associations among soil properties, plant growth traits, and microbial community composition. The lower-left panel shows Pearson’s correlation coefficients among soil variables (EC, pH, TN, NO_3_^-^-N, NH_4_^+^-N) and plant traits (plant height, fresh weight, dry weight), with colors indicating the direction and magnitude of correlations. The right panel shows Mantel test results linking soil variables to genus-level community composition, with edge color indicating Mantel p-value categories and edge width indicating Mantel r. These analyses are correlation-based and intended to summarize associational patterns.CK, LS, and MS represent control, low salinity, and moderate salinity treatments, respectively.

The Mantel tests indicated that variation in genus-level microbial community composition was associated with variation in soil physicochemical properties across samples (Mantel r = 0.60, *p* = 0.005; **Figure 9**). In the Pearson correlation matrix, plant growth traits (plant height, fresh weight, and dry weight) were positively correlated with total nitrogen (TN) and NO_3_^-^-N, but negatively correlated with NH_4_^+^-N, whereas EC and pH were negatively correlated with biomass traits (**Figure 9**).

When Mantel associations were examined by treatment group, LS and MS displayed more detected links (*p* < 0.05) than CK, with pH, EC, and TN showing relatively stronger associations with community composition (**Figure 9**). Overall, these results suggest that shifts in soil salinity-related properties and nitrogen status are accompanied by coordinated changes in microbial community structure, while plant growth traits covary with soil nitrogen forms. Given the low replication, these correlation-based results are interpreted as exploratory associations rather than evidence of causality.

## 4 Discussion

### 4.1 Effects of salinity on soil physicochemical properties and nitrogen transformation

Salinity markedly altered soil physicochemical properties and soil inorganic nitrogen status, consistent with characteristic features of saline soils. Notably, although MS received a higher salt addition than LS, EC measured at harvest (30 Sep 2024) was slightly higher in LS. This pattern may reflect not only the initial salt dose but also in-season salt transport and redistribution driven by irrigation.

Under regular irrigation, salts can migrate with soil water flow, and leaching may move soluble ions downward, causing the salt content in the sampled layer at harvest to deviate from a linear dose–response relationship (Wang et al., 2024; He et al., 2025). In addition, because the salt source included SO_4_²^-^, sulfate may react with Ca²^+^ in soil solution and promote precipitation of CaSO_4_ phases (e.g., gypsum), thereby reducing the dissolved-ion pool and EC, potentially more strongly under higher sulfate inputs (Van Driessche et al., 2019; Reiss et al., 2021; Ziegenheim et al., 2020). These mechanistic explanations remain speculative given that EC was assessed only at harvest; future work with time-series EC/major-ion measurements (e.g., Cl^-^, SO_4_²^-^, Na^+^, Ca²^+^, Mg²^+^) and leachate monitoring would be required to test them.

Soil pH increased significantly from CK to LS and further to MS, indicating progressive alkalinization under salinity. This increase may be related to Na^+^-associated alkalinization processes and exchange reactions that promote OH^-^ accumulation. Although legumes can acidify the root-zone via organic acid exudation to facilitate nutrient acquisition (He et al., 2020), this buffering capacity may be weakened under salinity stress, which could contribute to the observed pH increase (Su et al., 2022).

Salinity also shifted soil nitrogen status. Total N increased from CK to MS, which could reflect reduced plant N uptake or altered microbial turnover under saline conditions. NO_3_^-^-N was significantly elevated in MS, whereas CK and LS did not differ, suggesting that nitrate accumulation became more pronounced only under moderate salinity in this pot system. In contrast, NH_4_^+^-N was lower under salinity treatments than in CK, consistent with changes in ammonium retention and/or transformation under more alkaline conditions. These shifts in nitrate-to-ammonium balance likely reflect a combination of plant uptake and microbial processes and could also be influenced by NH_4_^+^ volatilization under higher pH (Min et al., 2014) and by salinity-related changes in leaching dynamics (Amini et al., 2016). In addition, salinity-induced inhibition of alfalfa nodulation has been reported (Aslani Borj et al., 2022) and may contribute to altered N inputs and nitrogen form distributions, although nodulation was not quantified in the present experiment.

### 4.2 Biphasic response of alfalfa growth to salinity

Alfalfa growth traits responded differentially across the salinity gradient, with shoot elongation and biomass accumulation showing distinct sensitivities. Plant height reached its highest mean value under low salinity (LS) and was significantly higher than under moderate salinity (MS), whereas the difference between LS and CK was not statistically significant. This pattern is consistent with the possibility that mild salinity coincided with maintained shoot elongation capacity in this experiment. Potential explanations include osmotic adjustment and stress signaling processes reported in other systems (e.g., proline-associated responses) (Kong et al., 2020), although these mechanisms were not directly measured here. Root-derived organic acids may also influence the root-zone chemical environment and could alleviate early constraints on growth (Fan et al., 2023). Under MS, plant height was numerically lower than CK and significantly lower than LS, consistent with stronger ionic stress and Na^+^-related disruption of K^+^ uptake constraining growth processes (Zhang et al., 2010), although direct ion measurements in plant tissues would be required to confirm this interpretation.

In contrast to plant height, biomass accumulation was more consistently reduced under salinity. Both LS and MS had significantly lower fresh and dry biomass than CK, while differences between LS and MS were not significant, indicating that biomass production was sensitive to salinity even when height differences were modest. The decline in fresh weight likely reflects osmotic constraints on plant water status, whereas reduced dry matter suggests limitations on carbon assimilation and growth efficiency. These patterns are consistent with reported salinity-associated impacts on photosynthesis (e.g., chloroplast damage and reduced Rubisco activity) (Noori et al., 2018) and ionic imbalance effects on protein synthesis and enzyme function (Li et al., 2010). Alfalfa can adjust biomass allocation (e.g., root:shoot ratio) under salinity (Liu et al., 2011), which may buffer some traits; however, the overall biomass reduction suggests that any compensation was insufficient to offset stress effects under the conditions tested.

### 4.3 Adaptive responses of microbial community structure and functional genes to salinity

In this pot experiment, salinity was associated with shifts in root-zone microbial community composition (β-diversity), whereas α-diversity metrics showed limited treatment effects. Shannon and Simpson indices at the genus and species levels did not differ among treatments, and the clearest α-diversity signal was observed for the KEGG-entry Simpson index. This pattern is consistent with the possibility that salinity influences functional evenness more detectably than taxonomic evenness at the tested sequencing depth. Such observations align with previous reports that salinity can reshape microbial composition and activity (Sunita et al., 2020), while functional redundancy may buffer diversity responses at broader functional levels (Debray et al., 2022; Wahab et al., 2023). Recent syntheses, including a meta-analysis across rhizosphere studies, similarly highlight salinity as a major driver of root-associated microbiome assembly across diverse plant systems (Abdelfadil et al., 2024);.

At the taxonomic level, several dominant genera exhibited treatment-associated abundance tendencies (e.g., lower relative abundance of *Nocardioides* and higher relative abundance of *Bradyrhizobium* and *Sediminibacterium* under salinity). However, none of the top 10 genera differed significantly among treatments after FDR correction; therefore, genus-level differences should be interpreted cautiously as trend-level patterns. Even so, the observed directions are compatible with the concept that salinity can act as an environmental filter through osmotic stress and ion toxicity, resulting in taxon-specific responses (Chen et al., 2024; Reginato et al., 2021). Moreover, mixed-salt inputs (NaCl and Na_2_SO_4_) may exert ion-specific effects on soil processes and biota (Irakoze et al., 2021; Reginato et al., 2021), potentially contributing to heterogeneous genus-level tendencies. Reports of salt-tolerant rhizobia (including *Bradyrhizobium*) supporting legume performance under salinity provide a plausible context for the observed tendency of *Bradyrhizobium* in this dataset (Dong et al., 2017), although symbiotic performance was not directly assessed here.

For nitrogen-cycling categories and KEGG metabolic pathways, heatmaps showed treatment-associated patterning in row-wise z-score profiles, with LS exhibiting distinct profiles for some functions relative to CK and MS. Importantly, formal tests on the underlying (non–z-score) relative abundance values did not yield significant between-treatment differences after FDR correction, indicating limited statistical support for pathway-specific claims. Accordingly, these functional results are best interpreted as coordinated, subtle trends in metagenome-inferred functional potential that may motivate targeted validation (e.g., process measurements, enzyme assays, or transcript-based analyses), rather than confirmatory evidence for shifts in specific nitrogen transformation pathways. Within these constraints, the observed patterns remain broadly consistent with prior work suggesting salinity effects on nitrogen transformation guilds (Ouni et al., 2014; Zhu et al., 2023) and stress-response strategies (e.g., transport and regulatory systems) in salt-affected environments (Waheed et al., 2022; Shilev, 2020). Overall, the present results are compatible with the view that environmental filtering and plant–microbe interactions may contribute to community reorganization under salinity (Quiroga et al., 2017; Zhang et al., 2019; Dastogeer et al., 2020; Yu et al., 2021), while highlighting that many apparent differences warrant validation in larger-scale studies given the low replication and multiple-testing correction (Nuccio et al., 2013).

### 4.4 Regulatory strategies for root-zone microbial-plant interactions under salinity

The observed covariation between salinity-related soil properties (e.g., EC) and plant biomass is consistent with widely reported salinity effects on plant water relations and ion balance. Mild salinity can sometimes coincide with short-term physiological adjustment (e.g., osmolyte accumulation and ion compartmentalization), whereas stronger or prolonged salinity is frequently linked to disrupted Na^+^/K^+^ homeostasis and growth inhibition (Kumar et al., 2021; Bano et al., 2021). In our dataset, plant growth traits also covaried with soil nitrogen forms, suggesting that nitrogen availability/status may be relevant to alfalfa performance under the tested conditions. Recent reviews also emphasize that plant–microbe interactions in the rhizosphere can modulate plant ion balance and stress responses under salinity, supporting the use of integrative soil–plant–microbiome frameworks for hypothesis generation (Ren et al., 2026).

Previous studies have reported that plant-associated symbioses (e.g., AMF) and beneficial microbial assemblages can be linked to improved nutrient acquisition and stress tolerance, and that root exudation may shape microbial community assembly under abiotic stress (Diagne et al., 2020; Hajihashemi et al., 2020; Liu et al., 2020). In contrast, weaker or inconsistent relationships involving NH_4_^+^–N could reflect multiple non-exclusive processes, including salinity impacts on nitrification, plant N uptake preference, and reduced NH_4_^+^ availability under higher pH due to volatilization losses (Mansour and Hassan, 2022; Kumar et al., 2021). Within this conceptual framework, enrichment of salt-tolerant taxa with stress-adaptive traits may accompany increases in EC, while mycorrhizal symbiosis and root-zone signaling processes have been proposed to support osmotic regulation and water balance (Sharma et al., 2019; Ramadhani and Widawati, 2020). Collectively, these literature-supported mechanisms provide hypotheses that are broadly consistent with the correlation patterns observed here and offer a conceptual basis for interpreting coordinated soil–microbe–plant responses along a salinity gradient (Hu et al., 2018; He et al., 2021a, He et al., 2021b, He et al., 2022).

Importantly, the present study provides associational evidence linking plant traits with soil chemistry and microbial functional profiles. Demonstrating causal mediation would require additional experiments (e.g., microbial inoculation, sterilized soil reconstitution, or targeted manipulation of key functions), ideally with larger replication and field-scale validation.

### 4.5 Limitations and future validation

Several limitations should be considered. First, biological replication was low (n = 3 per treatment), which limits statistical power-particularly for multivariate analyses and functional comparisons after FDR correction-and increases uncertainty around subtle effects. Second, this was a pot experiment with restricted rooting volume and simplified water–salt dynamics; therefore, extrapolation to field-scale saline soils should be made cautiously. Third, shotgun metagenomics infers functional potential rather than directly measuring nitrogen transformation rates; additional process-based measurements (e.g., N transformation rates, enzyme activities, or metatranscriptomics) are needed to verify whether inferred functional trends translate into realized biogeochemical changes. Future studies with larger replication and field validation across saline gradients will be important to confirm the observed soil–microbiome–plant associations and evaluate their agronomic relevance.

## 5 Conclusions

This study presents an integrative, metagenomics-enabled framework linking salinity-driven root-zone soil chemistry changes with alfalfa performance and microbiome functional potential in a controlled pot system. Overall, the results support our hypotheses that salinity is associated with (i) shifts in microbial community composition (β-diversity) more strongly than α-diversity, (ii) coordinated trend-level changes in metagenome-inferred nitrogen- and stress-related functional potential (most not significant after FDR correction), and (iii) covariation among soil salinity-related properties, microbiome features, and plant growth.

From a systems perspective, salinity altered root-zone chemical conditions and inorganic N balance while coinciding with reduced alfalfa biomass and microbiome reorganization. These coupled patterns suggest that saline forage systems may benefit from management and monitoring strategies that integrate soil EC/pH and inorganic N status with root-zone microbiome indicators.

Future work should validate these associations under field conditions with larger replication and process-based measurements (e.g., N transformation rates, enzyme activities, or metatranscriptomics) to determine whether metagenome-inferred trends translate into realized functional activity relevant to sustainable management of salt-affected soils.

## Data Availability

Primary data on which the analyses presented in this paper are all based, are provided as **Supplementary Materials**. Raw sequencing data are available in NCBI Sequence Read Archive (SRA) under Bioproject PRJNA1399466.
