# Supplementary material for: Salinity gradients alter root-zone soil microbiome structure and nitrogen-related functional potential in alfalfa (Medicago sativa L.): a pot experiment
Source: Front Plant Sci. 2026 Feb 13;17:1753229. doi: 10.3389/fpls.2026.1753229 (PMC12946031; doi:10.3389/fpls.2026.1753229)
Supplement: Supplementary file 10 [file Table8.docx]

**Gene abundance calculation**

1. **Definition**
   Ab(S) is the estimated abundance of gene *g* in sample *S*, combining uniquely mapped and multi-mapped reads and normalized by gene length.
2. **Equations**

$\boldsymbol{Ab}\left( \boldsymbol{S} \right)\mathbf{=}\boldsymbol{Ab}\left( \boldsymbol{U} \right)\boldsymbol{+Ab}\left( \boldsymbol{M} \right)$ (1)

$\boldsymbol{Ab}\left( \boldsymbol{U} \right)\mathbf{=}\sum_{\boldsymbol{i=1}}^{\boldsymbol{M}} \boldsymbol{1/l}$ (2)

$\boldsymbol{Ab}\left( \boldsymbol{M} \right)\mathbf{=}\sum_{\boldsymbol{i=1}}^{\boldsymbol{M}} \boldsymbol{(Co*1)/l}$ (3)

$\boldsymbol{Co}\mathbf{=}\frac{\boldsymbol{Ab(U)}}{\sum_{\boldsymbol{i=1}}^{\boldsymbol{N}} \boldsymbol{Ab(}\boldsymbol{U}_{\boldsymbol{i}}\boldsymbol{)}}$ (4)

1. **Variable definitions (bullet list)**

- **Ab(S)**: total gene abundance estimate in sample S
- **Ab(U)**: contribution from **uniquely mapped** reads
- **Ab(M)**: contribution from **multi-mapped** reads after proportional allocation
- **l**: gene length (bp) used for length normalization
- **M**: number of reads (or read assignments) contributing to the term (state clearly: unique or multi-mapped, depending on the equation)
- **N**: number of candidate genes to which a multi-mapped read can align
- **Co**: allocation coefficient; multi-mapped reads are distributed across candidate genes in proportion to their Ab(U)
